# Supplementary material for: Healthy Lifestyle Management of Pediatric Obesity with a Hybrid System of Customized Mobile Technology: The PediaFit Pilot Project
Source: Nutrients. 2021 Feb 16;13(2):631. doi: 10.3390/nu13020631 (PMC7919673; doi:10.3390/nu13020631)
Supplement: Supplementary file 1 [file nutrients-13-00631-s001.pdf]

nutrients

Supplementary Tables

Table S1. Changes of anthropometric parameters of PediaFit 1.2 vs PediaFit 1.1 patients.

Variable	IG 3 MONTHS, mean (SD)			IG 6 MONTHS, mean (SD)		
	PediaFit 1.2	PediaFit 1.1	P Value	PediaFit 1.2	Pediafit1.1	P Value
BMI Kg/m²	-2,2 (0,9)	-2,36 (1,29)	0,6	-4,6 (1,8)	-2,99 (2,96)	0,17
BMI ZS	-1,29 (1,3)	-0,28 (0,15)	0,01	-1,8 (0,7)	-0,33 (0,3)	0,08
Ex WC%	-30,9 (23,83)	-36,11 (38,12)	0,000	-34,19 (27,07)	-28,89 (43,65)	0,70
Ex NC%	-38,41 (40,23)	-59,58 (42,20)	0,18	-57,18 (44,52)	-54,031(67,19)	0,89
SBP mmHg	-14,03 (8,5)	-9,58 (9,87)	0,16	-24,64 (25,7)	-6,25 (14,33)	0,07
DBP mmHg	-11,58 (15,0)	-3,63 (7,10)	0,11	-2,37 (17,26)	-1,88 (10,67)	0,64
AN grade	-0,8 (0,5)	-0,41 (0,51)	0,03	-1,0 (0,6)	-0.75(0,89)	0,4

AN: Acanthosis Nigricans decrease; BMI: body mass index; BMI zs: z-score BMI; CG = Control Group; Ex WC: excess waist circumference by 95° percentile; Ex NC: Excess Neck circumference by 95° percentile; DBP: Diastolic Blood pressure; IG= Intervention Group; SBP: Systolic blood pressure.

Table S2. Comparison of the changes of lifestyle parameters PediaFit 1.2 vs PediaFit 1.1.

Variable	3 MONTHS, mean SD			6 MONTHS, mean SD		
	IG 1.2	IG 1.1	P Value	IG 1.2	IG 1.1	P Value
SuD (ml/week)	-587,0 (367,8)	-673,5 (487,5)	0,6	-860,0 (586)	-718,0 (504,2)	0.5
ScreenT (min/day)	-83,8 (93,0)	-45,0 (101,05)	0,22	-118,7 (100,2)	-81,4 (95,9)	0,3
Sleep (h/night)	0,6 (0,9)	0,3 (0,5)	0,02	1,18 (1,5)	-0,58 (1,65)	0,02
F&V (portion/die)	1,18 (1,6)	1,25 (1,09)	0,5	2,57 (1,1)	2,03 (1,2)	0,5
PA (min/week)	71,85 (118,0)	0,76 (12,55)	0,03	112,2 (113,1)	11,2 (63,59)	0,01

CG= Control Group; F&V: fruits and vegetables; IG= Intervention Group; PA: physical activity; ScreenT: screen time; SuD: Sugary drinks.
